# Chemosensitivity testing of small cell lung cancer using the MTT assay.

**DOI:** 10.1038/bjc.1991.16

**Published:** 1991-01

**Authors:** B. G. Campling, J. Pym, H. M. Baker, S. P. Cole, Y. M. Lam

**Affiliations:** Department of Oncology, Queen's University, Kingston, Ontario, Canada.

## Abstract

A simple colorimetric test, the MTT assay, has been adapted for chemosensitivity testing of human small cell lung cancer cell lines, and fresh tumour samples. Optimal conditions for clinical chemosensitivity testing were determined using established SCLC lines. Nineteen different chemotherapeutic agents were tested, and sixteen of them were found to be cytotoxic in this assay system. The drug sensitivity of a panel of 16 SCLC cell lines was measured and compared. There was very little intraexperiment variation, but the interexperiment variation was significant. Cell lines which were derived from patients who had not received chemotherapy at the time the cell line was established were more sensitive (to all but one of the drugs) than lines derived from treated patients, and the differences were statistically significant for two of the drugs. One cell line, NCI-H209, which was derived from an untreated patient, stood out as being the most sensitive or among the most sensitive to all of the drugs tested. Another cell line, H69AR, which is a multidrug resistant subline of the cell line NCI-H69, was the most resistant to many of the natural product drugs tested. Multiple drug chemosensitivity testing was performed on eight fresh tumour samples from SCLC patients (five pleural effusions, one lymph node, and two primary tumours). It was possible to perform chemosensitivity testing on all of the clinical samples in which sufficient tumour cells were available. The drug sensitivity of the clinical samples was, in most cases, within the same range as for the cell lines. Since this assay is very rapid and simple to perform, it may have practical applications in clinical drug sensitivity testing of human tumours.


Br. J. Cancer (1991), 63, 75-83 © Macmillan Press Ltd., 1991

### Chemosensitivity testing of small cell lung cancer using the MTT assay

B.G. Campling[1], J. Pym[2], H.M. Baker[1], S.P.C. Cole[3] & Y.-M. Lam[4]

[1]*Departments of Oncology and Medicine;* [2]*Department of Surgery;* [3]*Departments of Oncology, Pathology, and Pharmacology and Toxicology;* [4]*Department of Community Health and Epidemiology; Queen's University, Kingston, Ontario, Canada K7L 3N6.*

**Summary** A simple colorimetric test, the MTT assay, has been adapted for chemosensitivity testing of human small cell lung cancer cell lines, and fresh tumour samples. Optimal conditions for clinical chemosensitivity testing were determined using established SCLC lines. Nineteen different chemotherapeutic agents were tested, and sixteen of them were found to be cytotoxic in this assay system. The drug sensitivity of a panel of 16 SCLC cell lines was measured and compared. There was very little intraexperiment variation, but the interexperiment variation was significant. Cell lines which were derived from patients who had not received chemotherapy at the time the cell line was established were more sensitive (to all but one of the drugs) than lines derived from treated patients, and the differences were statistically significant for two of the drugs. One cell line, NCI-H209, which was derived from an untreated patient, stood out as being the most sensitive or among the most sensitive to all of the drugs tested. Another cell line, H69AR, which is a multidrug resistant subline of the cell line NCI-H69, was the most resistant to many of the natural product drugs tested. Multiple drug chemosensitivity testing was performed on eight fresh tumour samples from SCLC patients (five pleural effusions, one lymph node, and two primary tumours). It was possible to perform chemosensitivity testing on all of the clinical samples in which sufficient tumour cells were available. The drug sensitivity of the clinical samples was, in most cases, within the same range as for the cell lines. Since this assay is very rapid and simple to perform, it may have practical applications in clinical drug sensitivity testing of human tumours.

Lung cancer has become an epidemic in North America, accounting for over 100,000 deaths annually. Small cell lung cancer comprises 20 to 25% of these cases. Although the prognosis in SCLC has improved considerably with the use of chemotherapy, and response rates are high, only a minority of patients are cured of the disease. A therapeutic plateau has now been reached, and new approaches are required to improve the results of treatment with currently available chemotherapeutic agents.

The results of empiric selection of chemotherapeutic drugs for treatment of individual patients with lung cancer could be improved by the use of a practical predictive assay of drug sensitivity. To aid in making therapeutic decisions, such an assay should be simple, rapid, inexpensive, and available to most patients. The clonogenic assay of Hamburger and Salmon (1977) has been used for predictive chemosensitivity testing, and positive clinical correlations have been found in a variety of tumour types (Browman *et al.*, 1983; Park *et al.*, 1980; Von Hoff *et al.*, 1981), including lung cancer (Shimizu *et al.*, 1981). However, the assay is highly labour-intensive, results are not available for 2-3 weeks, and numerous technical and theoretical problems remain (Browman *et al.*, 1986; Selby *et al.*, 1983; Twentyman, 1985).

As a result, there has been renewed interest in short term assays of drug sensitivity (Weisenthal & Lippman, 1985). The MTT assay is a simple colorimetric test of cell proliferation and survival which was developed by Mosmann (1983) and adapted by Cole (1986) and others for measuring chemosensitivity of human lung cancer cell lines. A number of groups have used this assay for drug sensitivity testing of human cell lines (Carmichael *et al.*, 1987, 1988; Finlay *et al.*, 1986; Park *et al.*, 1987; Twentyman & Luscombe, 1987), and it is now being used for screening new anticancer agents (Alley *et al.*, 1988, Ruben & Neubauer, 1987). It has also been used for chemosensitivity testing of fresh human leukaemia samples (Campling *et al.*, 1988; Pieters *et al.*, 1989; Twentyman *et al.*, 1989). In most instances, there is a close correlation between results of drug sensitivity testing using this assay and the clonogenic assay (Carmichael *et al.*, 1987; Wasserman & Twentyman, 1988). Because of its simplicity, the MTT assay has the potential to overcome a number of the problems encountered with other chemosensitivity assays which have prevented their routine use in clinical practice.

The purpose of the present investigation was to adapt the MTT assay for chemosensitivity testing of fresh human SCLC tumour samples. Initial experiments were performed using human SCLC cell lines in order to determine appropriate seeding cell densities, drug concentrations and drug incubation times. Once optimal conditions were determined, the chemosensitivity of a panel of SCLC cell lines was measured and compared. In addition, eight fresh tumour samples from SCLC patients were tested for their sensitivity to a broad range of chemotherapeutic agents. Although the MTT assay has been used extensively for chemosensitivity testing of human tumour cell lines, to the best of our knowledge, there have been no previous published reports of its use for chemosensitivity testing of human solid tumours.

### Materials and methods

### *Cell lines*

The cell lines used for this study are described in Table I. The source of the lines, and the treatment and response histories of the patients from whom the lines were derived are indicated in this table. Many of the lines were established in this laboratory and will be described in detail in a future publication. The cell lines NCI-H69, NCI-H128, and NCI-H209 were provided by Dr J. Minna, NCI-Navy Medical Oncology Branch, National Cancer Institute, Bethesda, Maryland (Carney *et al.*, 1985). H69AR is a multidrug resistant variant of NCI-H69, which was selected in Adriamycin (Mirski *et al.*, 1987). Cell line Mar was a gift from Prof. A. Neville, Ludwig Institute for Cancer Research, London, UK (Ibson *et al.*, 1987). Cell line SHP-77, established by Fisher and Paulson (1978), was obtained from Dr Jorgen Fogh, Sloan Kettering Institute for Cancer Research, New York. Cell lines RG-1 and MM-1 were established by Dr W.E.C. Bradley, Institut du Cancer de Montréal, and characterised in this laboratory. All of the lines are SCLC cell lines with the possible exception of three lines, BK-T, HG-E and SHP-77, in which there is some controversy as to the histologic diagnosis. BK-T is either SCLC or an atypical carcinoid tumour, and HG-E is either an intermediate cell variant of

Correspondence: B.G. Campling, The Ontario Cancer Treatment and Research Foundation, Kingston Regional Cancer Centre, King St West, Kingston, Ontario, Canada K7L 2V7.
Received 20 November 1989; and in revised form 3 September 1990.

**Table I** Cell lines used for chemosensitivity studies

| *Line* | *Clinical history* | *Source (reference)* |
|---|---|---|
| AD-A | Needle aspirate of subcutaneous lump in a patient who had progressive disease after multi-agent chemotherapy. | This laboratory |
| BK-T | Primary tumour resected from a patient who subsequently received combination chemotherapy and is alive and well several years later. | This laboratory |
| LG-T | Lymph node biopsy from a patient with limited SCLC who subsequently had a complete response to chemotherapy (but has since recurred). | This laboratory |
| HG-E | Pleural effusion from a patient with extensive metastatic disease who died shortly afterwards and never received chemotherapy. | This laboratory |
| JO-E | Pleural effusion from a patient who had recurred following a complete response to chemotherapy. | This laboratory |
| WL-E | Pleural effusion from a patient who had been treated with chemotherapy with a minimal response. | This laboratory |
| JN-M | Bone marrow from a patient with otherwise limited SCLC, who subsequently had a partial response to chemotherapy and radiotherapy. | This laboratory |
| SH-A | Needle aspirate of a supraclavicular node from a patient who had previously had a complete response to chemotherapy, and subsequently responded to further chemotherapy. | This laboratory |
| RG-1 | Pericardial effusion from a patient who had recurred after chemotherapy for limited stage SCLC. | W.E.C. Bradley. Characterized in this laboratory |
| MM-1 | Pleural effusion from a patient who had recurrent disease after combination chemotherapy for limited stage SCLC. | W.E.C. Bradley. Characterized in this laboratory |
| NCI-H69 | Pleural effusion from a patient who had received chemotherapy. | J.D. Minna (Carney *et al.*, 1985) |
| H69AR | Multidrug-resistant variant of NCI-H69 selected in adriamycin. | S.P.C. Cole (Mirski, Gerlach & Cole, 1987) |
| NCI-H128 | Pleural effusion from a patient who had received chemotherapy. | J.D. Minna (Carney *et al.*, 1985) |
| NCI-H209 | Bone marrow from an untreated patient. | J.D. Minna (Carney *et al.*, 1985) |
| Mar | Derived from an untreated patient. | A. Neville (Ibson *et al.*, 1987) |
| SHP-77 | Primary tumour from a patient who had not received chemotherapy. | Fisher & Paulson (Fisher & Paulson, 1978) (obtained from J. Fogh) |

SCLC or large cell anaplastic carcinoma (Campling *et al.*, submitted). SHP-77 is considered a large cell variant of SCLC (Fisher & Paulson, 1978).

All cell lines were maintained at 37°C in a humidified atmosphere with 5% $CO_2$. The medium used was RPMI 1640 medium supplemented with hydrocortisone (10 nM, Sigma), insulin (10 $\mu g\ ml^{-1}$, Sigma), transferrin (10 $\mu g\ ml^{-1}$, Sigma), estradiol (10 nM, Sigma), and sodium selenite (30 nM) (HITES medium) supplemented with 2.5% FBS (SS-HITES). Antibiotics were not routinely used. The cells were fed or subcultured at least once weekly.

### *Fresh tumour samples*

Seventeen clinical samples from SCLC patients were received in the laboratory. These specimens were taken for diagnostic or therapeutic indications, and samples were sent for drug sensitivity testing with the informed consent of the patients. The type and quantity of the samples are shown in Table II. The solid tumour specimens were disaggregated using a wire mesh. The effusion samples were centrifuged and the cells washed in RPMI medium. For all of the fresh tumour samples, viable cells were separated from red cells and debris using a Ficoll Hypaque density gradient, and the cells were washed and resuspended in SS-HITES supplemented with 50 $\mu g\ ml^{-1}$ of gentamycin. Cytocentrifuge preparations were made from each of the specimens to determine the proportion of tumour cells present.

Drug sensitivity studies were performed on eight of these samples (numbers 1, 3, 7, 8, 13, 14, 16 and 17). Sample 1 consisted of pleural fluid from a previously untreated patient with extensive SCLC. The patient died a few days after the first chemotherapy treatment. Sample 3 was a supraclavicular lymph node biopsy taken from a patient with a lung mass. The biopsy revealed a diagnosis of SCLC. The patient was subsequently treated with radiotherapy, and chemotherapy

**Table II** Clinical samples from SCLC patients

| *Sample number* | *Type of sample** | *Volume of sample†* | *Cell count‡* | *Cytology* | *Number of drugs tested* |
|---|---|---|---|---|---|
| 1 | PE | 200 ml | $2 \times 10^7$ | Almost entirely SCLC | 9 |
| 2 | PerE | 150 ml | $< 10^6$ | Mostly lymphocytes, occasional cells suspicious of SCLC | – |
| 3 | LN | 1.5 $cm^3$ | $3.4 \times 10^7$ | Almost entirely SCLC | 11 |
| 4 | PE | 200 ml | $< 10^6$ | Mostly lymphocytes | – |
| 5 | PE | 400 ml | $< 10^6$ | Mostly lymphocytes | – |
| 6 | PE | 100 ml | $< 10^6$ | Mostly lymphocytes | – |
| 7 | PE | 1000 ml | $2.6 \times 10^7$ | 70% of cells SCLC, rest lymphocytes and occasional mesothelial cells | 9 |
| 8 | PE | 500 ml | $5.6 \times 10^7$ | Predominantly SCLC, occasional lymphocytes and mesothelial cells | 9 |
| 9 | PE | 60 ml | $< 10^6$ | Occasional cluster of SCLC | – |
| 10 | PE | 500 ml | $< 10^6$ | Mostly lymphocytes | – |
| 11 | PE | 230 ml | $1.0 \times 10^6$ | Lymphocytes outnumbered SCLC 5:1 | – |
| 12 | PE | 2150 ml | $< 10^6$ | Mostly lymphocytes, occasional clumps of SCLC | – |
| 13 | PE | 1000 ml | $4.8 \times 10^7$ | Almost entirely SCLC | 12 |
| 14 | PE | 450 ml | $5.7 \times 10^7$ | Almost entirely SCLC | 10 |
| 15 | PE | 45 ml | $< 10^6$ | Mostly lymphocytes | – |
| 16 | Prim | 2.0 $cm^3$ | $2.4 \times 10^7$ | Almost entirely SCLC | 11 |
| 17 | Prim | 2.5 $cm^3$ | $3.4 \times 10^7$ | Almost entirely SCLC | 11 |

*Pleural effusion, PE; pericardial effusion, Per E; lymph node, LN; primary tumour, Prim. †Preparation of the samples is outlined in the Materials and methods section. ‡Total number of SCLC cells in the sample.

(cyclophosphamide, adriamycin and vincristine (CAV)), and had a minor response. Sample 7 consisted of pleural fluid from a patient with SCLC who had progressed after an initial response to chemotherapy (CAV alternating with VP-16 and cisplatin (VP/CP)). Sample 8 was pleural fluid from a patient with extensive SCLC who had progressed on chemotherapy (one cycle of CAV followed by one cycle of VP/CP). The patient subsequently had a partial response to radiotherapy and further chemotherapy. Sample 13 consisted of pleural fluid from a patient with extensive SCLC who had progressed after one cycle of chemotherapy (CAV). The patient refused further therapy. Sample 14 was pleural fluid from a patient who had developed progressive disease several months after a partial response to six cycles of chemotherapy (CAV alternating with VP/CP). Samples 16 and 17 were resected primary tumours from patients with limited SCLC. Both patients are currently receiving chemotherapy.

### *Drugs*

Most of the drugs were obtained from the Kingston General Hospital Pharmacy, already dissolved in the appropriate diluent for infusion. These drugs included Adriamycin, epirubicin, daunomycin, menogaril, mitoxantrone, VP-16, VM-26, vincristine, vinblastine, cisplatin, carboplatin, mitomycin C, m-amsacrine, nitrogen mustard, and methotrexate. Procarbazine and CCNU were obtained in capsule form, from the same source, and the contents of the capsule were dissolved in sterile medium. Melphalan was obtained from Sigma, and was dissolved in acidified ethanol. 4-Hydroperoxycyclophosphamide was obtained from Dr M. Colvin, The Johns Hopkins Hospital, Baltimore, MD. Most of the drugs were stored at − 20°C in aliquots at 20 times the desired final concentration. Cisplatin, VP-16, VM-26, and mitoxantrone were stored at room temperature, and vincristine was stored at 4°C.

### *MTT assay*

*Cell preparation* All of the cell lines grew as floating aggregates with the exception of SHP-77, which was loosely adherent to plastic. To disaggregate the cells, they were centrifuged, resuspended, and drawn up and down three times through a 21 gauge needle. Cells were resuspended in SS-HITES, and dispensed into 96 well microtitre plates (Linbro 76-032-05) at the desired cell density using a 100 μl multichannel pipette.

*Cell density* The effect of seeding cell densities on absorbance values was determined over a range of cell densities on one of the cell lines (BK-T), and on one of the clinical samples which consisted entirely of tumour cells (sample 13). To determine the relative contribution to the absorbance readings of contaminating normal cells which may be present in clinical tumour samples, fresh human peripheral blood mononuclear cells, obtained by Ficoll Hypaque density gradient centrifugation, were tested in the same manner. Cells were plated in duplicate 96 well microtitre plates at densities ranging from $10^3$ to $5 \times 10^5$ cells per well, and each cell density was tested in four to eight wells. Absorbances were determined (after addition of MTT (see below)) either immediately or after 48 h. For all comparative chemosensitivity studies with cell lines and with fresh tumour samples, a cell density of $10^5$ cells/well was used.

### *Chemosensitivity studies*

*Drug dilutions* Drugs were added approximately 16 h after the cells were plated, diluted in SS-HITES to twice the desired final concentration. One hundred microlitres of drug solution were added to wells containing 100 μl of cell suspension. Serial dilutions of drug were tested in concentrations ranging from 100 μg ml$^{-1}$ to 0.1 μg ml$^{-1}$. Controls included wells with cells without drug, and wells with the highest concentrations of drug without cells. Each drug concentration was tested in quadruplicate.

*Chemosensitivity assay duration* Using the cell line NCI-H69, varying durations of drug exposure were tested, ranging from 1 to 3 days. The following drugs were tested: Adriamycin, cisplatin, menogaril, mitoxantrone, nitrogen mustard and VP-16. For comparative chemosensitivity studies, a drug exposure time of 48 h was used.

*Effect of cell density on cytotoxicity* It is difficult to obtain highly accurate cell counts using SCLC cell lines because the cells often form tight aggregates. Because of concerns that drug induced cytotoxicity might be affected by the seeding cell density (Chambers *et al.*, 1984; Ohnuma *et al.*, 1986), the cytotoxicity of several drugs was compared at different cell densities. Cell lines BK-T and NCI-H69 were used for these studies. Plates were set up at densities of $6 \times 10^4$, $8 \times 10^4$, $10^5$ and $2 \times 10^5$ cells per well. Serial dilutions of the following drugs were tested at each cell density: adriamycin, cisplatin, daunomycin, epirubicin, mitomycin C, nitrogen mustard, vinblastine, VP-16, and 4-hydroperoxycyclophosphamide.

*Comparative chemosensitivity studies* Dose response curves were generated for the 16 cell lines with each of the 16 active drugs. A standard cell density of $10^5$ cells per well, and a drug incubation time of 48 h were used for these studies. Each of the cell lines was tested at least once with each of the drugs, and many of the lines were tested on multiple occasions, up to seven times with the same drug. Eight of the fresh clinical samples had sufficient tumour cells for multiple drug chemosensitivity testing, and nine to twelve drugs were tested on each of these eight samples.

### *Development of the plates*

MTT (Sigma) was dissolved in PBS to a concentration of 2 mg ml$^{-1}$. After the desired incubation time with drug, 100 μl of medium was removed and 25 μl of MTT solution was added to each well. After 6 h incubation at 37°C, 100 μl of 1 N HCl:isopropanol (1:24) was added to each well and mixed vigorously using a multichannel pipette. To increase the solubilisation of the formazan crystals, the plates were then incubated at 37°C for 1 h as described (Campling *et al.*, 1988). Absorbance values at 570 nm were determined on a Dynatech MR600 microtitre plate reader.

### *Data analysis and statistics*

Data were entered on a Digital Microvax II minicomputer using a form-based system, stored in a relational database (Vax Rdb), and retrieved with a simple query language, Datatrieve. Custom software was used to calculate means and standard deviations for each drug concentration and generate dose response curves for each drug, to be displayed on either a graphics terminal or a laser printer for hardcopy.

The dose response curves were normalised so that the baseline absorbance with no added drug was given a value of one. Two summary statistics were used for describing and comparing the dose response curves, namely the $IC_{50}$ (the concentration of drug which caused 50% reduction in absorbance compared to baseline values), and the area under the dose response curve (AUC). The $IC_{50}$ was derived by fitting a fourth degree polynomial regression to the data, and the AUC was calculated between drug concentrations of 0 and 100 μg ml$^{-1}$ by the trapezoidal method (Moon 1980). The same concentration range was used for all of the drugs.

The intraexperiment variation (or repeatability) of the assay was assessed by determining the standard deviation of the AUC. This was done by generating a family of dose response curves by connecting the four data points at each drug concentration to each of the four data points at all of the other drug concentrations. The AUC for each of these curves was determined, and the mean AUC and standard deviation calculated. The interexperiment variation (or reproducibility) of the assay was determined by calculating the mean and standard deviation for the AUC of repeated tests with the same drug and cell line. All statistical analyses were performed using the SAS statistical package (SAS Institute Inc., 1985).

*Clinical correlation*

The cell lines were classified according to the treatment status of the patients from whom the lines were established. This information is provided in Table I. It can be seen that cell lines BK-T, LG-T, HG-E, NCI-H209, Mar, and SHP-77 were derived from untreated patients, and cell lines AD-A, JO-E, WL-E, JN-M, SH-A, RG-1, MM-1, NCI-H69, and NCI-H128 were derived from treated patients. The cell line H69AR was excluded from this analysis, since it had undergone *in vitro* selection for drug resistance. The mean AUC for all of the cell lines from untreated patients was determined for each of the drugs and compared to the mean AUC for the cell lines from treated patients. Analysis of variance was applied to determine the statistical significance of the differences between cell lines from untreated patients and from treated patients for each of the drugs. A similar comparison was made for the clinical samples.

### Results

Sixteen of the 19 drugs tested were cytotoxic in this assay. Procarbazine, methotrexate, and CCNU did not cause cytotoxicity and the reasons for this are unclear at the present time. It was possible to perform chemosensitivity testing with multiple drugs on all of the cell lines and on eight of the clinical samples (all of the samples in which sufficient tumour cells were obtained).

*Cell density*

The relationship of cell density to absorbance is shown in Figure 1. It can be seen that there is a linear relationship over the range of densities tested. The absorbance of this particular clinical sample was greater than that of the cell line, BK-T. However, as discussed below, the baseline absorbances of the clinical samples were quite variable. The absorbance of the peripheral blood mononuclear cells was much less than either the tumour sample or the cell line at all cell densities. For example, at $10^5$ cells per well, the absorbance of the lymphocytes was approximately one tenth that of the fresh tumour sample. In the experiment shown, MTT was added immediately after the cells had been plated. In a second experiment, MTT was added after 48 h incubation, and the results were essentially the same (not shown).

*Assay duration*

The effects of different drug exposure times were determined, and the results with six different drugs are shown in Figure 2. On the basis of this data and other results with clinical samples (Campling *et al*., 1988), a drug incubation time of two days was selected for comparative chemosensitivity studies. This exposure time was sufficient to produce cytotoxicity with the 16 active drugs tested, and short enough to minimise the variable effects of cell proliferation and cell death over the assay period, an important consideration when the assay is applied to fresh tumour samples. For the three drugs which were not cytotoxic in this assay, namely procarbazine, methotrexate and CCNU, cytotoxicity was not seen even with drug incubation times of up to 1 week.

*Effect of cell density on cytotoxicity*

At the cell densities tested ($6 \times 10^4$, $8 \times 10^4$, $10^5$, and $2 \times 10^5$ cells/well), there were no significant differences in cytotoxicity with the nine drugs tested (data not shown). Because of the difficulty in obtaining an accurate cell count with SCLC samples due to cell clumping, it is important to know that the assay is reproducible over this range of cell densities.

*Data analysis*

The $IC_{50}$ and AUC were used to summarise and compare the dose response curves. It was not always possible to determine the $IC_{50}$ since in some cases there was very little cytotoxicity even at the highest drug concentrations, and in other cases there was greater than 50% cytotoxicity at the lowest drug concentrations. In a few instances, the $IC_{50}$ was poorly estimated since there was stimulation of growth at the lowest drug concentrations (Vichi & Tritton, 1989). For these reasons, the AUC has been used to express the cytotoxicity data. The details of the statistical analysis are presented elsewhere (Lam *et al*., 1989). As outlined in this paper, there was a linear relationship between the log of the $IC_{50}$ and the AUC.

*Comparative chemosensitivity studies*

The results of comparative chemosensitivity testing of the 16 cell lines with the 16 different drugs are shown in Figure 3. The cell lines have been rank ordered according to AUC. When a particular cell line has been tested more than once with the same drug, the standard deviations for repeat determinations are shown, and the number of times the experiment has been repeated is indicated as a measure of interexperiment variation, or reproducibility of the assay. The intraexperiment variation of the AUC's was quite low, with coefficients of variation generally less than 4%.

It can be seen that one particular cell line, NCI-H209, stood out as being the most sensitive, or among the most sensitive to all of the drugs tested. This included drugs which are part of the multidrug resistance phenotype (Gerlach *et al*., 1986), such as the anthracyclines and vinca alkaloids, as well as other drugs such as alkylating agents and platinum analogues. This cell line was tested on multiple occasions from 1986 to 1989, and the pattern of sensitivity has remained stable.

Another cell line, H69AR, which was selected for resistance to adriamycin and is known to be multidrug resistant (Mirski *et al*., 1987), was the most resistant to many of the natural product drugs, including Adriamycin, epirubicin, vinblastine, vincristine, and VP-16, and was among the most resistant to daunomycin and menogaril. It is interesting that this line was resistant to one of the alkylating agents tested, namely melphalan. Surprisingly, it did not appear to be resistant to mitoxantrone or VM-26, and it was not resistant to amsacrine, cisplatin, carboplatin, mitomycin C, nitrogen mustard, or 4-hydroperoxycyclophosphamide. Cell line NCI-H69, the parent line from which H69AR was derived, and which was established from a patient who had received chemotherapy, was not outstandingly sensitive or resistant to the majority of the 16 drugs tested.

*Fresh tumour samples*

As outlined in Table II, 17 samples from SCLC patients were received in the laboratory, and there were sufficient tumour cells for multiple drug chemosensitivity testing in eight of these samples. The cytological composition of the samples is shown in this table. It can be seen that all three of the solid tumour samples consisted of virtually a pure population of tumour cells in adequate numbers. There were sufficient tumour cells in only five of the 14 effusion samples. In six of the effusion samples there were no definite malignant cells present. The majority of the contaminating cells in these effusions were lymphocytes. Although there were occasional macrophages and mesothelial cells in some of the samples, they did not make up a significant percentage of the total cell population. Thus, the results presented here represent the drug sensitivity of tumour cells, and not that of non malignant cells in the specimens.

The baseline absorbances for the clinical samples (i.e. absorbance of untreated control wells plated at $10^5$ cells/well, with MTT added after 2 days) were as follows: Sample 1:

**Figure 1** A linear regression analysis showing the relationship between cell density and absorbance, for one clinical sample (sample 13), cell line BK-T, and peripheral blood mononuclear cells (PBM's). The dotted lines indicate 95% confidence limits.

0.300, sample 3: 0.240, sample 7: 0.730, sample 8: 0.580, sample 13: 1.480, sample 14: 1.401, sample 16: 0.180, and sample 17: 0.653. Thus, it can been seen that the extent of dye reduction by clinical samples of SCLC is highly variable.

The AUC's for the eight patient samples are shown in Table III. The range of AUC's for the entire panel of cell lines is also indicated in this table. It can be seen that, in most cases, the drug sensitivity of the clinical samples was within the same range as for the cell lines.

*Clinical correlation*

As shown in Table IV, the mean AUC for cell lines derived from treated patients was consistently greater than that for untreated patients, for all of the drugs except one (vincristine). The differences were statistically significant for two of the drugs, namely carboplatin, and nitrogen mustard. To the best of our knowledge, none of the patients from whom the cell lines were derived ever received either of these drugs.

A similar comparison was made for the clinical samples, and there were no significant differences between samples obtained from treated *vs* untreated patients. However, there

**Figure 2** Effect of different drug exposure times from 24 to 72 h for six different drugs (adriamycin, cisplatin, menogaril, mitoxantrone, nitrogen mustard, and VP-16). All of these experiments were done using cell line NCI-H69. Error bars are omitted for clarity. Drug exposure times were 24 h ●· · · · · ·●, 48 h ☆------☆ and 72 h Δ———Δ.

**Figure 3** AUC's for the 16 SCLC cell lines tested with 16 drugs. In those cases in which the experiment has been performed more than once, standard deviations are indicated. The number of determinations is indicated in parentheses underneath the name of the cell line. The coefficients of variation for individual experiments were generally less than 4%. The abbreviations used for the cell lines on these histograms are as follows: AD-A, AD; BK-T, BK; LG-T, LG; HG-E, HG; JO-E, JO; WL-E, WL, JN-M, JN; SH-A, SH; RG-1, RG; MM-1, MM; NCI-H69, H69; H69AR, AR; NCI-H128, 128; NCI-H209, 209; Mar, MAR; SHP-77, S77.

**Table III** AUC's for clinical samples from SCLC patients *untreated, †treated at time of testing. (N.T. = not tested)

| Drug | Range for cell lines | Area under dose response curve Sample 1* | Sample 3* | Sample 7† | Sample 8† | Sample 13† | Sample 14† | Sample 16* | Sample 17* |
|---|---|---|---|---|---|---|---|---|---|
| Adriamycin | 7.4–47.0 | 13.2 ± 2.0 | 34.1 ± 2.8 | 23.7 ± 2.2 | 15.3 ± 0.7 | 22.3 ± 0.6 | 26.7 ± 1.2 | 25.9 ± 1.6 | 15.9 ± 0.6 |
| Carboplatin | 22.5–86.5 | 53.2 ± 3.0 | 56.4 ± 3.1 | 86.9 ± 3.3 | 78.0 ± 2.5 | 95.5 ± 2.2 | 82.5 ± 3.0 | 83.0 ± 5.1 | 88.9 ± 1.2 |
| Cisplatin | 7.5–43.0 | 12.9 ± 2.5 | N.T. | 44.9 ± 0.9 | 10.8 ± 0.3 | 50.9 ± 1.3 | 28.9 ± 0.7 | 29.6 ± 1.7 | 19.3 ± 2.7 |
| Epirubicin | 2.6–57.0 | 28.1 ± 2.4 | 36.5 ± 2.7 | 17.8 ± 0.3 | 8.3 ± 0.4 | 13.3 ± 0.6 | 23.3 ± 0.5 | 17.7 ± 1.6 | 15.5 ± 1.2 |
| Menogaril | 3.5–17.3 | 18.2 ± 2.8 | 18.7 ± 2.1 | N.T. | N.T. | 7.7 ± 0.3 | N.T. | 11.8 ± 1.6 | 11.8 ± 0.4 |
| Mitoxantrone | 14.8–28.1 | 36.6 ± 8.0 | 9.2 ± 3.3 | 18.4 ± 1.1 | 8.2 ± 0.6 | 10.3 ± 0.5 | 23.4 ± 1.1 | 29.5 ± 1.8 | 17.0 ± 1.6 |
| Nitrogen mustard | 5.9–29.8 | 8.4 ± 0.6 | 28.1 ± 2.6 | N.T. | N.T. | 13.8 ± 0.3 | 28.3 ± 1.0 | 18.7 ± 1.5 | 15.6 ± 0.4 |
| Vincristine | 30.7–84.1 | 54.1 ± 3.4 | 68.3 ± 9.2 | 60.0 ± 2.0 | 84.9 ± 2.2 | 55.4 ± 1.0 | 72.0 ± 1.6 | 59.7 ± 2.8 | 68.5 ± 2.0 |
| VM26 | 5.0–32.9 | N.T. | 17.5 ± 2.0 | N.T. | 28.0 ± 1.6 | 9.4 ± 0.4 | 27.0 ± 0.9 | 34.0 ± 1.6 | 45.8 ± 1.9 |
| VP16 | 33.0–86.6 | 53.9 ± 1.6 | 54.3 ± 4.0 | 81.7 ± 2.6 | 120.6 ± 4.7 | 57.9 ± 1.0 | 95.6 ± 2.8 | 59.4 ± 2.2 | 67.3 ± 2.5 |
| 4H-Cyclophosphamide | 11.1–55.2 | N.T. | 25.0 ± 3.1 | 50.9 ± 0.7 | 90.9 ± 1.5 | 74.9 ± 2.2 | 92.2 ± 3.0 | 30.1 ± 1.0 | 88.1 ± 2.4 |

The clinical history of the patients in whom drug sensitivity testing was performed is indicated in the Materials and methods section.

**Table IV** Comparison of mean AUC of cell lines derived from untreated *vs* treated patients

| Drug | Comparison of cell lines from untreated vs treated patients Mean AUC – untreated | Mean AUC – treated | P value |
|---|---|---|---|
| Adriamycin | 24.3 | 28.2 | 0.30 |
| Amsacrine | 13.4 | 19.2 | 0.13 |
| Carboplatin | 50.5 | 63.0 | 0.05* |
| Cisplatin | 25.9 | 29.4 | 0.32 |
| 4H-Cyclophosphamide | 27.3 | 30.0 | 0.67 |
| Daunomycin | 9.7 | 16.2 | 0.17 |
| Epirubicin | 19.1 | 21.8 | 0.48 |
| Melphalan | 30.8 | 35.0 | 0.38 |
| Menogaril | 9.8 | 11.1 | 0.45 |
| Mitomycin C | 24.9 | 28.6 | 0.48 |
| Mitoxantrone | 18.4 | 24.0 | 0.07 |
| Nitrogen mustard | 12.7 | 18.5 | 0.02* |
| Vinblastine | 35.6 | 41.7 | 0.45 |
| Vincristine | 56.9 | 49.3 | 0.28 |
| VM26 | 20.3 | 24.3 | 0.53 |
| VP16 | 42.5 | 50.4 | 0.18 |

*Indicates statistical significance.

were only four patients in each group, and it was not possible to make any comment about the response status of the untreated patients, since one of them died before treatment could be given, two of them had their tumours resected, and were thus not assessable for response to chemotherapy, and one had a very minimal response to subsequent treatment.

### Discussion

The use of a predictive assay of drug sensitivity could have an impact on the management of patients with lung cancer. In fact, a recently reported clinical trial in extensive SCLC comparing empiric selection of chemotherapy to selection based on the results of chemosensitivity testing suggests that response rates may be improved by this approach (Gazdar *et al.*, 1990). The clonogenic assay has proven to be of limited practical use since drug sensitivity testing can be performed in only a minority of lung cancer cases (De Vries *et al.*, 1987; Kitten *et al.*, 1982), although improved culture techniques have resulted in higher success rates (Kanzawa *et al.*, 1987).

The major advantages of the MTT assay are its speed and simplicity. Because most steps are automated, it is possible to test multiple drugs, each at several concentrations. The automated data analysis is essential in view of the large amounts of data that can be generated. Since results are available within 3 days, such information may be of value in clinical therapy. Furthermore, a short assay duration will minimise the variable effects of cell proliferation and cell death over the assay period.

In order to perform testing of multiple drugs, relatively large numbers of cells are required. However, the test as we describe it requires no more cells than the clonogenic assay, or the DiSC assay of Weisenthal (Weisenthal *et al.*, 1983), which, when applied to lung cancer, requires $2 \times 10^5$ cells per data point (De Vries *et al.*, 1987).

It has been argued that the potential application of this assay to clinical chemosensitivity testing is limited (Carmichael *et al.*, 1987; 1988). In fact, it would likely not be possible to perform the assay as described by others in the majority of clinical samples, because of the low seeding cell densities commonly used. The importance of having the cells in exponential growth phase during the assay period has been emphasised. However, the majority of samples derived from patients will not be growing exponentially.

Carmichael *et al.* (1987) reported high coefficients of variation (15% on octuplicate determinations) when the MTT assay was used for sensitivity testing of cell lines that grow in suspension, such as SCLC. However, in the present study we found that the coefficients of variation on quadruplicate determinations were usually less than 4%. There are a number of possible explanations for this discrepancy. In the Carmichael study, DMSO was used to solubilise the formazan crystals. We have found that DMSO increased absorbance values, but also increased the intraexperimental variation. Futhermore, when DMSO is used, most of the medium must be removed from the wells prior to the addition of DMSO. It would be difficult to avoid removing some cells in the process, and this could contribute to the higher standard deviations observed. Finally, since low seeding cell densities are used, there could be differential cell growth in the wells during the assay period, thus increasing the coefficients of variation.

Although the intraexperiment variation reported here is less than that of other investigators, the interexperiment variation is a significant problem which needs to be considered when interpreting chemosensitivity results, and may be a limiting factor if the assay is to be applied in the clinical setting. We believe that the interassay variability is more likely to be a function of the cell lines than an inherent problem with the MTT assay. The cell lines were tested repeatedly over a period of 3 years. While no trends towards either increasing or decreasing drug sensitivity were noted over this period of time, it is possible that the drug sensitivity may have been unstable. Other potential causes of interassay variation are differences in growth rates between the cell lines, and variations in cell cycle parameters which cannot be completely controlled. These problems are more likely to produce significant artifacts when longer assay durations are employed. This was one of the considerations in our selection of a short drug incubation time. It should also be noted that it is much easier to quantitate interexperimental variation using the MTT assay than with other assays of cytotoxicity because of the ease with which multiple repeat assays can be performed.

Contamination of tumour specimens with infiltrating non-malignant cells is a potential problem with the MTT assay, as with most other short-term assays of cytotoxicity. However, the clinical samples that we tested had very minimal contamination with non-tumour cells. The predominant non-malignant cells in the samples received to date have been lymphocytes. Furthermore, we have shown that peripheral blood mononuclear cells (which include lymphocytes) reduce the tetrazolium dye much less than SCLC tumour cells. Thus, the chemosensitivity results of the eight clinical samples presented here represent the drug sensitivity of tumour cells. Overgrowth of fibroblasts has not occurred with the assay described here, likely because of the short drug incubation time, and the use of selective medium. Contamination of tumour samples with non-malignant cells may prove to be a more significant problem with other tumour types. However, if large numbers of contaminating non-malignant cells are present, it should be possible to remove them using a variety of cell separation procedures.

The MTT assay described here is conceptually somewhat different than that developed by Cole (1986) for chemosensitivity testing of human lung cancer cell lines. Cole's method uses lower seeding cell densities and a longer drug incubation period. Thus, it measures a combination of drug-induced cytotoxicity and inhibition of cell growth. In the present study, a shorter drug incubation time was used, measuring primarily cytotoxicity. Tumour samples obtained directly from patients cannot necessarily be expected to proliferate in tissue culture.

The relative resistance to natural product drugs of H69AR compared to its parent cell line NCI-H69 appears to be less striking than originally reported by Mirski *et al.* (1987). There are two reasons for this discrepancy. Firstly, we have expressed our data as AUC's, whereas Mirski *et al.* expressed their data in terms of $IC_{50}$'s. We have shown that the AUC relates most closely to the logarithm of the $IC_{50}$ (Lam *et al.*, 1989). Thus, relative resistance values using the $IC_{50}$ may appear much greater than when using the AUC. The second reason is that the MTT assay performed here is quite different than that used by Mirski *et al.*, as discussed above. Using the assay as described here we found that H69AR was cross resistant to the same spectrum of drugs as found by Mirski *et al.*, with the exception of mitoxantrone. We did not find H69AR to be cross resistant to this particular drug, whereas Mirski *et al.*, found it to be cross resistant. The reasons for this discrepancy are unclear at the present time.

Sixteen SCLC cell lines were used for comparative chemosensitivity studies. These include lines established from patients with a spectrum of clinically drug sensitive and drug resistant disease. Only one of these lines (H69AR) had undergone *in vitro* selection for drug resistance. As well, although all of the cell lines are examples of SCLC, and were treated as such, they represent a pathologic spectrum, ranging from one cell line which was difficult to distinguish from an atypical carcinoid tumour, to one which was difficult to distinguish from large cell anaplastic carcinoma. From a clinical, pathological and biological point of view, SCLC is a heterogeneous disease. We believe that if one is to make clinically relevant conclusions regarding the spectrum of clinical drug responsiveness of SCLC cell lines, it is important to include a large number of cell lines representative of the complete pathologic and clinical spectrum of the disease.

The comparative chemosensitivity studies of these SCLC cell lines reveal some interesting patterns of drug sensitivity and resistance. In particular, one cell line, NCI-H209, stood out as the most sensitive to most of the drugs tested, including natural products, alkylating agents, and platinum compounds. The cell line H69AR, which had been selected in adriamycin, was the most resistant to a number of natural product drugs. Because it is feasible to test multiple drugs, this assay has the potential to reveal valuable information regarding the incidence of multidrug resistance in the clinical setting.

A number of investigators (Batist *et al.*, 1986; Carmichael *et al.*, 1988; Carney *et al.*, 1983; Ruckdeschel *et al.*, 1987; Tsai *et al.*, 1989) have found a close correlation between chemotherapy treatment status and the relative *in vitro* chemosensitivity of the cell line derived from that patient. We found that cell lines established from untreated patients were more sensitive to nearly all of the drugs than lines established from treated patients. However, the results were statistically significant for only two of the drugs, and the differences for most of the drugs were not striking.

It is not possible on the basis of data presented here to make any clinical correlations on clinical samples. It would also be premature to attempt to make any definitions of *in vitro* sensitivity and resistance on the basis of data from such a small number of patients. Studies currently in progress of clinical chemosensitivity testing in haematologic malignancies should give a definitive answer regarding clinical correlations using this assay.

In summary, we have shown that the MTT assay can be applied to *in vitro* chemosensitivity testing of SCLC lines and fresh clinical samples. While some technical problems remain, this assay may have potential applications for predictive chemosensitivity testing.

This research was supported by grants from the Cancer Research Society Inc., The Banting Research Foundation, the Clare Nelson Bequest of Kingston General Hospital, and the National Cancer Institute of Canada. We thank Dr W.S. Lofters and Dr A.A. Conlan for providing some of the clinical samples.

### References

ALLEY, M.C., SCUDIERO, D.A., MONKS, A. & 5 others (1988). Feasibility of drug screening with panels of human tumor cell lines using a microculture tetrazolium assay. *Cancer Res.*, **48**, 589.

BATIST, G., CARNEY, D., COWAN, K.H. & 4 others (1986). Etoposide (VP-16) and cisplatin in previously treated small-cell lung cancer: clinical trial and *in vitro* correlates. *J. Clin. Oncol.*, **4**, 982.

BROWMAN, G., GOLDBERG, J., GOTTLEIB, A.J. & 5 others (1983). The clonogenic assay as a reproducible *in vitro* system to study predictive parameters of treatment outcome in acute non-lymphoblastic leukemia. *Am. J. Hematol.*, **15**, 227.

BROWMAN, G.P., LEVINE, M.N. & ROBERTS, R.S. (1986). Bench-to-bedside research and the human tumor stem-cell assay-Bridging the credibility gap. *J. Clin. Oncol.*, **4**, 1730.

CAMPLING, B.G., PYM, J., GALBRAITH, P.R. & COLE, S.P.C. (1988). Use of the MTT assay for rapid determination of chemosensitivity of human leukemic blast cells. *Leukemia Res.*, **12**, 823.

CARMICHAEL, J., DEGRAFF, W.G., GAZDAR, A.F., MINNA, J.D. & MITCHELL, J.B. (1987). Evaluation of a tetrazolium based semi-automated colorimetric assay. I: assessment of chemosensitivity testing. *Cancer Res.*, **47**, 936.

CARMICHAEL, J., MITCHELL, J.B., DEGRAFF, W.G. & 5 others (1988). Chemosensitivity testing of human lung cancer cell lines using the MTT assay. *Br. J. Cancer*, **57**, 540.

CARNEY, D.N., GAZDAR, A.F., BEPLER, G. & 5 others (1985). Establishment and identification of small cell lung cancer cell lines having classic and variant features. *Cancer Res.*, **45**, 2913.

CARNEY, D.N., MITCHELL, J.B. & KINSELLA, T.J. (1983). *In vitro* radiation and chemotherapy sensitivity of established cell lines of human small cell lung cancer and its large cell morphological variants. *Cancer Res.*, **43**, 2806.

CHAMBERS, S.H., BLEEHEN, N.M. & WATSON, J.V. (1984). Effect of cell density on intracellular adriamycin concentrations and cytotoxicity in exponential and plateau phase EMT6 cells. *Br. J. Cancer*, **49**, 301.

COLE, S.P.C. (1986). Rapid chemosensitivity testing of human lung tumor cells using the MTT assay. *Cancer Chemother. Pharmacol.*, **17**, 259.

DE VRIES, E.G.E., MEIJER, C., MULDER, N.H. & POSTMUS, P.E. (1987). *In vitro* chemosensitivity of human lung cancer for vindesine. *Eur. J. Cancer Clin. Oncol.*, **23**, 55.

FINLAY, G.J., WILSON, W.R. & BAGULEY, B.C. (1986). Comparison of *in vitro* activity of cytotoxic drugs towards human carcinoma and leukaemia cell lines. *Eur. J. Cancer Clin. Oncol.*, **22**, 655.

FISHER, E.R. & PAULSON, J.D. (1978). A new *in vitro* cell line established from human large cell variant of oat cell lung cancer. *Cancer Res.*, **38**, 3830.

GAZDAR, A.F., STEINBERG, S.M., RUSSELL, E.K. & 7 others (1990). Correlation of *in vitro* drug sensitivity testing results with response to chemotherapy in extensive stage small cell lung cancer: a prospective clinical trial. *J. Natl Cancer Inst.*, **82**, 117.

GERLACH, J.H., KARTNER, N., BELL, D.R. & LING, V. (1986). Multidrug resistance. *Cancer Surveys*, **5**, 25.

HAMBURGER, A.W. & SALMON, S.E. (1977). Primary bioassay of human tumor stem cells. *Science*, **197**, 461.

IBSON, J.M., WATERS, J.J., TWENTYMAN, P.R., BLEEHEN, N.M. & RABBITTS, P.H. (1987). Oncogene amplification and chromosomal abnormalities in small cell lung cancer. *J. Cell Biochem.*, **33**, 267.

KANZAWA, F., MATSUSHIMA, Y., HAMBURGER, A.W. & 5 others (1987). Human tumor clonogenic assay for carcinoma of the lung II. Factors that influence colony formation in soft agar. *Oncology*, **44**, 150.

KITTEN, C.M., VON HOFF, D.D., BENNETT, E.V. & GROVER, F.L. (1982). Growth of lung cancer in a human tumor clonogenic system. *J. Thorac. Cardiovasc. Surg.*, **83**, 363.

LAM, Y.-M., PYM, J. & CAMPLING, B.G. (1989). Dose response analysis of chemosensitivity testing of small cell lung cancer using the MTT assay. *Am. Stat. Assoc., Proc. of the Biopharmaceutical Section*, 193.

MIRSKI, S.E.L., GERLACH, J.H. & COLE, S.P.C. (1987). Multidrug resistance in a human small cell lung cancer line selected in adriamycin. *Cancer Research*, **47**, 2594.

MOON, T.E. (1980). Quantitative and statistical analysis of the association between *in vitro* and *in vivo* studies. In *Cloning of Human Tumour Stem Cells*, Salmon, S.E. (ed.) p. 209. Alan R. Liss: New York.

MOSMANN, T. (1983). Rapid colorimetric assay for cellular growth and survival: application to proliferation and cytotoxicity assays. *J. Immunol. Methods*, **65**, 55.

OHNUMA, T., ARKIN, H. & HOLLAND, J.F. (1986). Effects of cell density on drug-induced cell kill kinetics *in vitro* (inoculum effect). *Br. J. Cancer*, **54**, 415.

PARK, C.H., AMARE, M., SAVIN, M.A., GOODWIN, J.W., NEWCOMB, M. & HOOGSTRATEN, B. (1980). Prediction of chemotherapy response in human leukemia using an *in vitro* chemotherapy sensitivity test on the leukemic colony-forming cells. *Blood*, **55**, 595.

PARK, J.-G., KRAMER, B.S., STEINBERG, S.M. & 4 others (1987). Chemosensitivity testing of human colorectal carcinoma cell lines using a tetrazolium-based colorimetric assay. *Cancer Res.*, **47**, 5875.

PIETERS, R., HUISMANS, D.R., LEYVA, A. & VEERMAN, A.J.P. (1989). Comparison of the rapid automated MTT-assay with a dye exclusion assay for chemosensitivity testing in childhood leukaemia. *Br. J. Cancer*, **59**, 217.

RUBEN, R.L. & NEUBAUER, R.H. (1987). Semiautomated colorimetric assay for *in vitro* screening of anticancer compounds. *Cancer Treatment Reports*, **71**, 1141.

RUCKDESCHEL, J.C., CARNEY, D.N., OIE, H.K., RUSSELL, E.K. & GAZDAR, A.F. (1987). *In vitro* chemosensitivity of human lung cancer cell lines. *Cancer Treatment Reports*, **71**, 697.

SAS INSTITUTE INC. (1985). *SAS User's Guide: Statistics*, Version 5 Edition. Cary, NC: SAS Institute Inc.

SELBY, P., BUICK, R.N. & TANNOCK, I. (1983). A critical appraisal of the 'Human tumor stem-cell assay'. *N. Engl. J. Med.*, **308**, 129.

SHIMIZU, E., SAIJO, N., KANAZAWA, F. & 9 others (1981). Correlation between drug sensitivity determined by clonogenic assay and clinical effect of chemotherapy in patients with primary lung cancer. *Gann*, **75**, 1030.

TSAI, C.M., LAI, S.L., IHDE, D.C. & 5 others (1989). Clinical response to combination chemotherapy in lung cancer is correlated with *in vitro* chemosensitivity but not with expression of the *mdr1* gene. *Proc. ASCO*, **8**, 247 (Abstr 965).

TWENTYMAN, P.R. (1985). Predictive chemosensitivity testing. Guest Editorial. *Br. J. Cancer*, **51**, 295.

TWENTYMAN, P.R., FOX, N.E. & REES, J.K.H. (1989). Chemosensitivity testing of fresh leukaemia cells using the MTT colorimetric assay. *Br. J. Haematol.*, **71**, 19.

TWENTYMAN, P.R. & LUSCOMBE, M. (1987). A study of some variables in a tetrazolium dye (MTT) based assay for cell growth and chemosensitivity. *Br. J. Cancer*, **56**, 279.

VICHI, P. & TRITTON, T.R. (1989). Stimulation of growth in human and murine cells by adriamycin. *Cancer Res.*, **49**, 2679.

VON HOFF, D.D., CASPER, H., BRADLEY, E., SANDBACK, J., JONES, D. & MAKUCH, R. (1981). Association between human tumor colony-forming assay results and response to chemotherapy. *Am. J. Med.*, **70**, 1027.

WASSERMAN, T.H. & TWENTYMAN, P.R. (1988). Use of a colorimetric microtiter (MTT) assay in determining the radiosensitivity of cells from murine solid tumors. *Int. J. Radiat. Oncol. Biol. Phys.*, **15**, 699.

WEISENTHAL, L.M. & LIPPMAN, M.E. (1985). Clonogenic and nonclonogenic *in vitro* chemosensitivity assays. *Cancer Treatment Rep.*, **69**, 615.

WEISENTHAL, L.M., MARSDEN, J.A., DILL, P.L. & MACALUSO, C.K. (1983). A novel dye exclusion method for testing *in vitro* chemosensitivity of human tumors. *Cancer Res.*, **43**, 749.


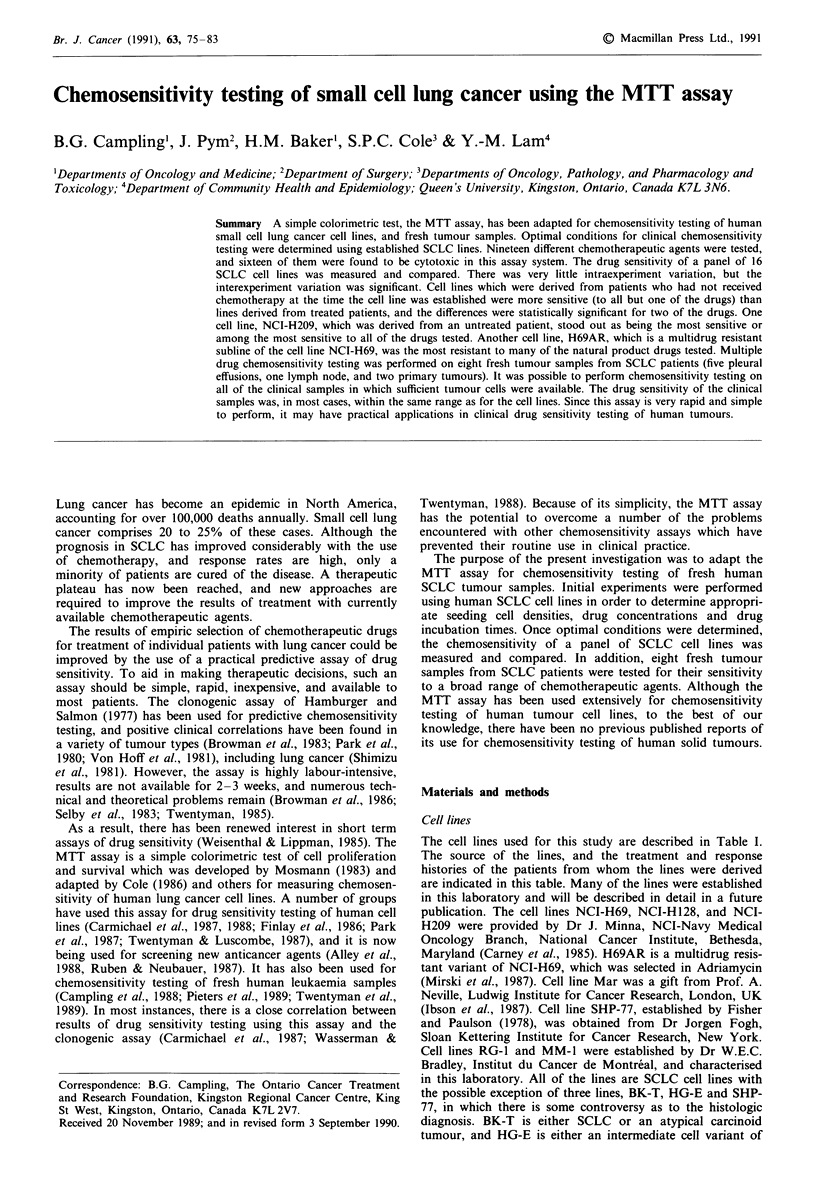

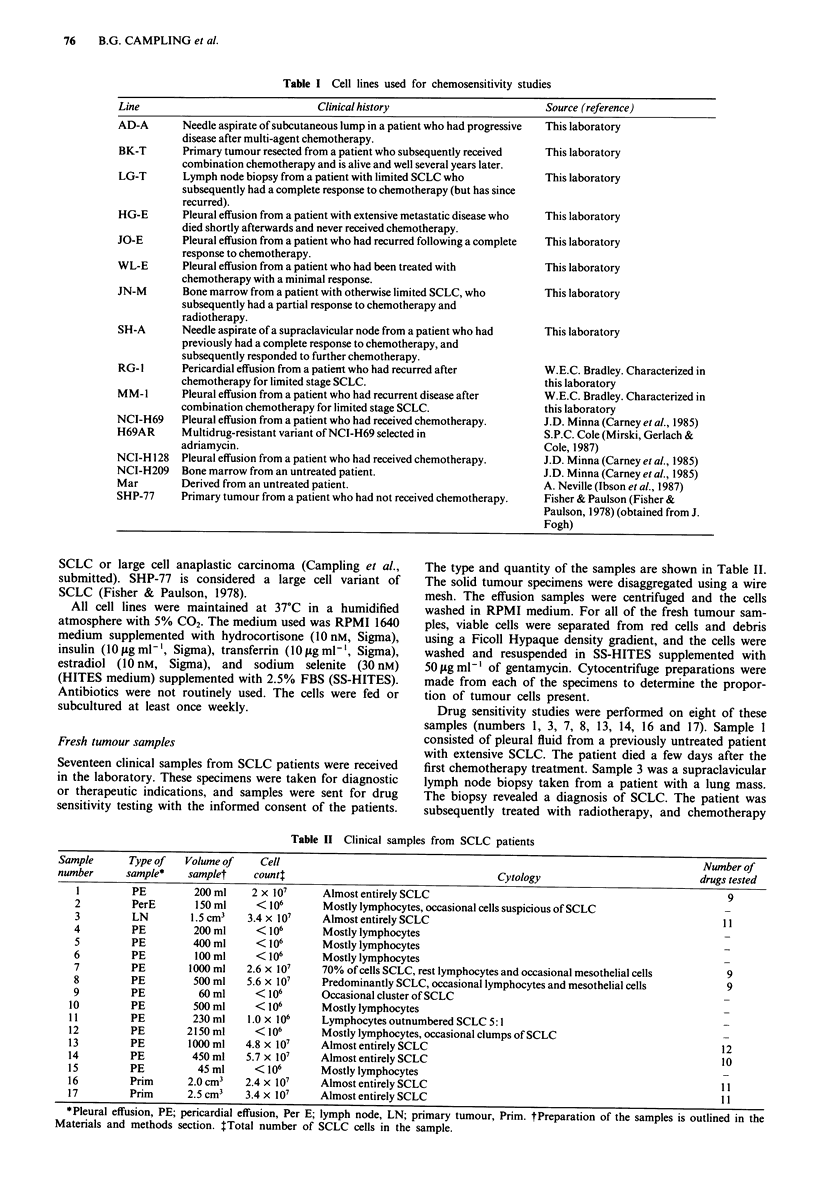

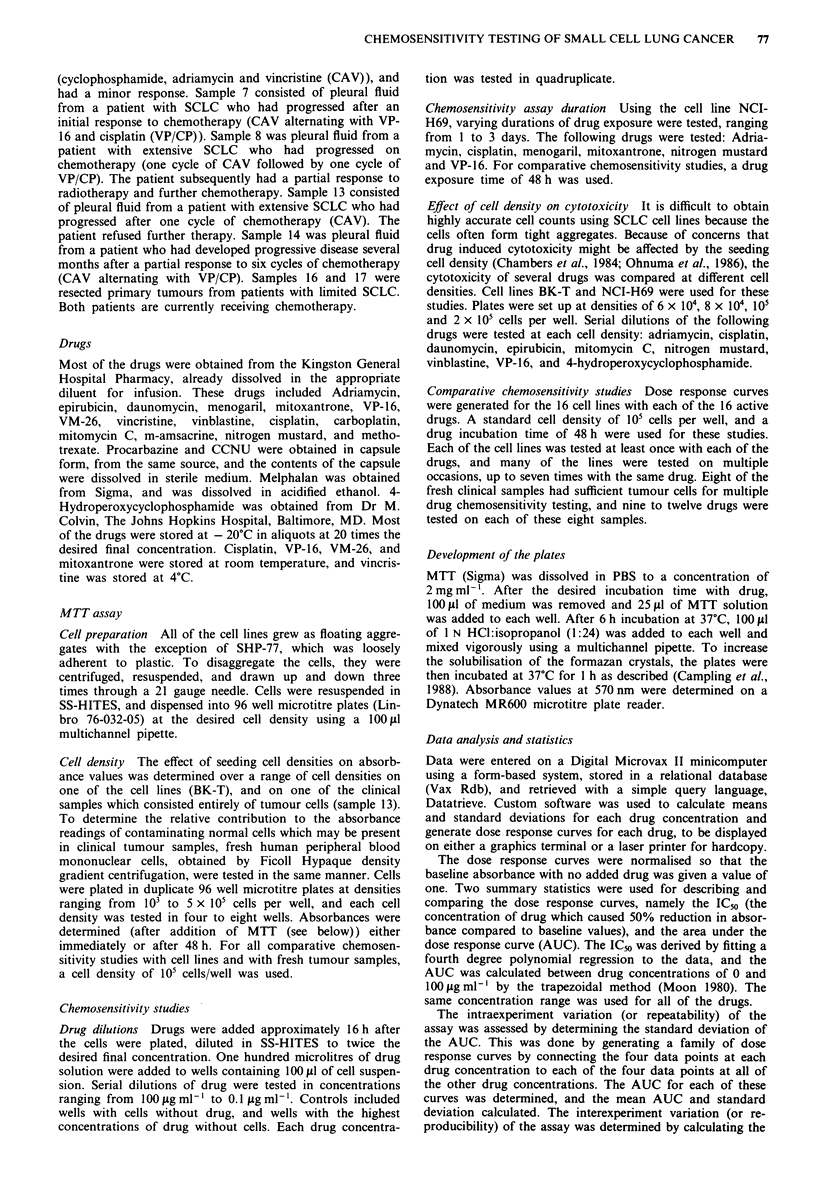

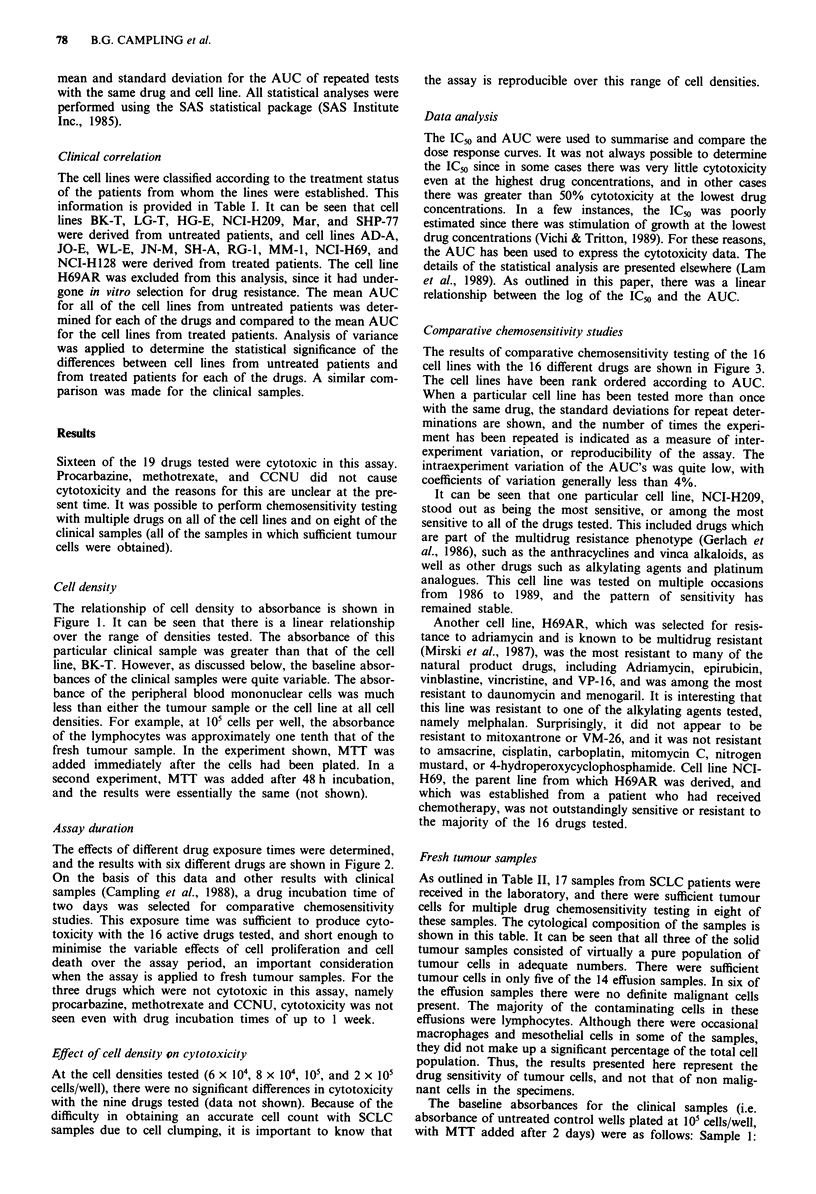

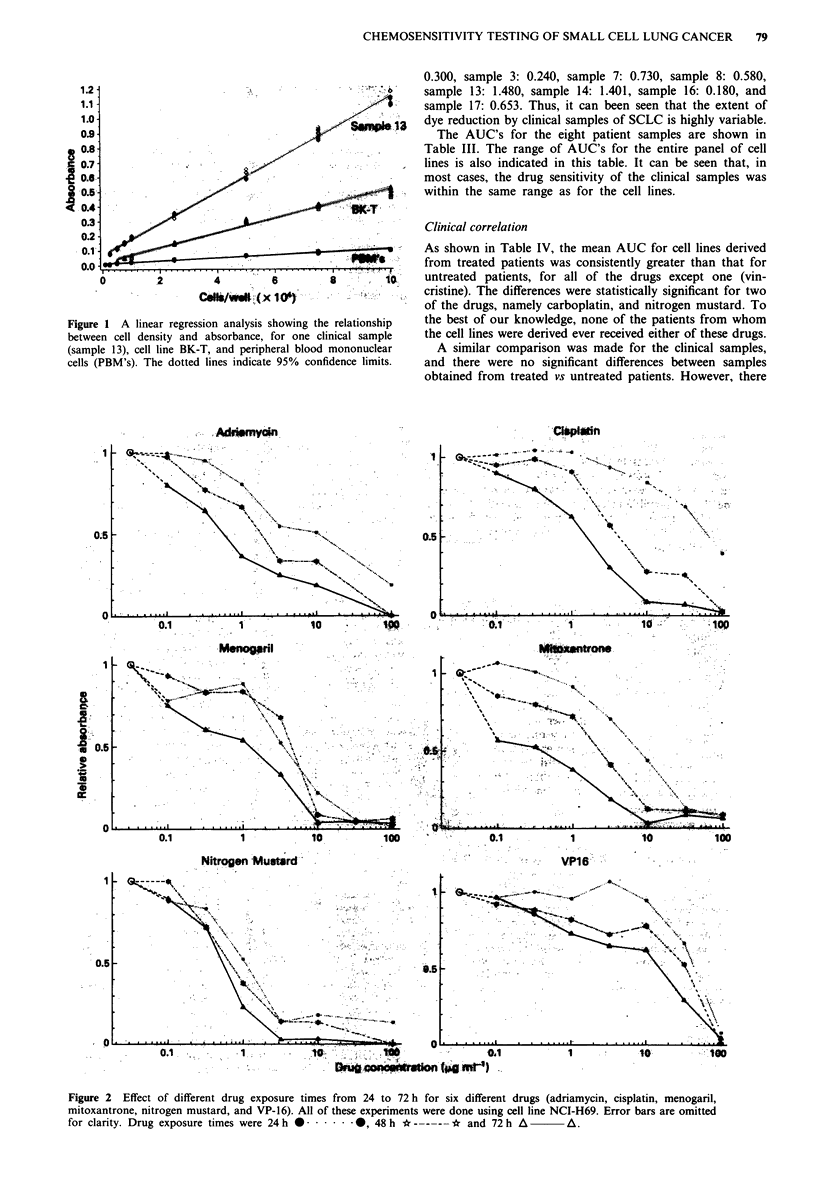

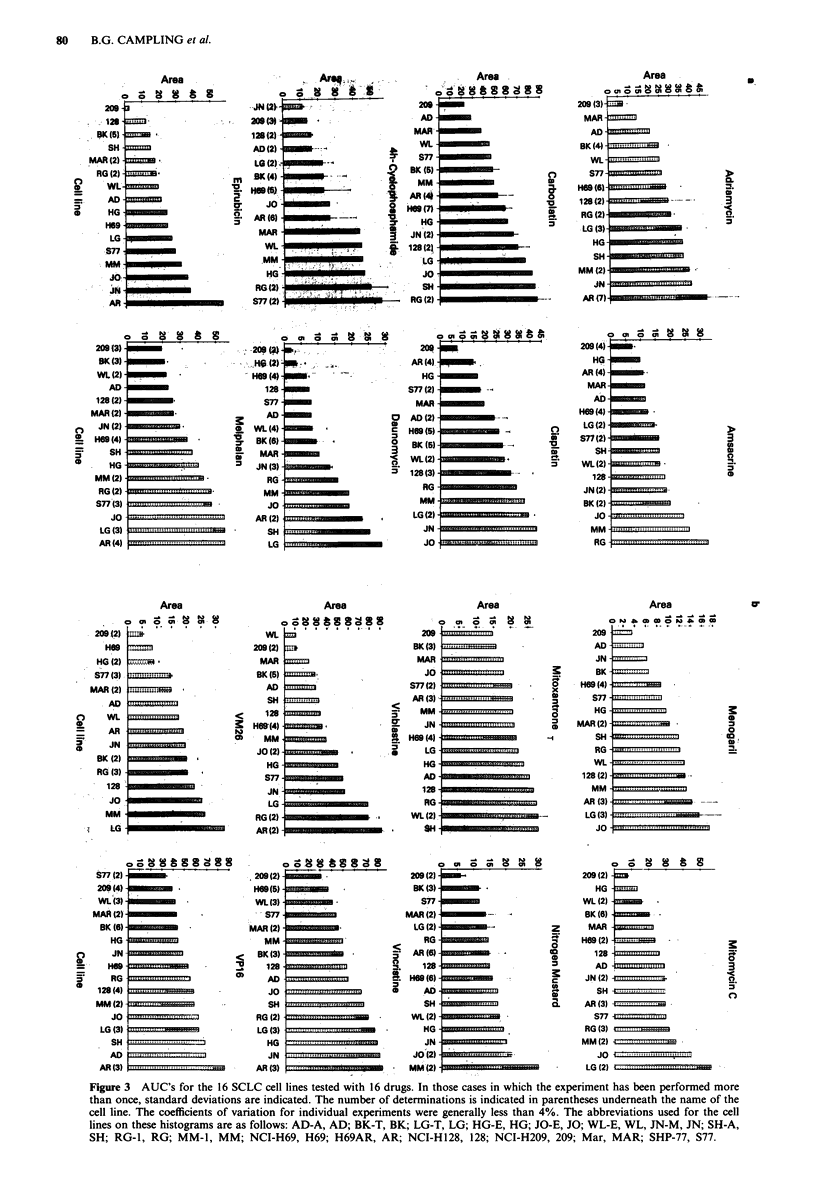

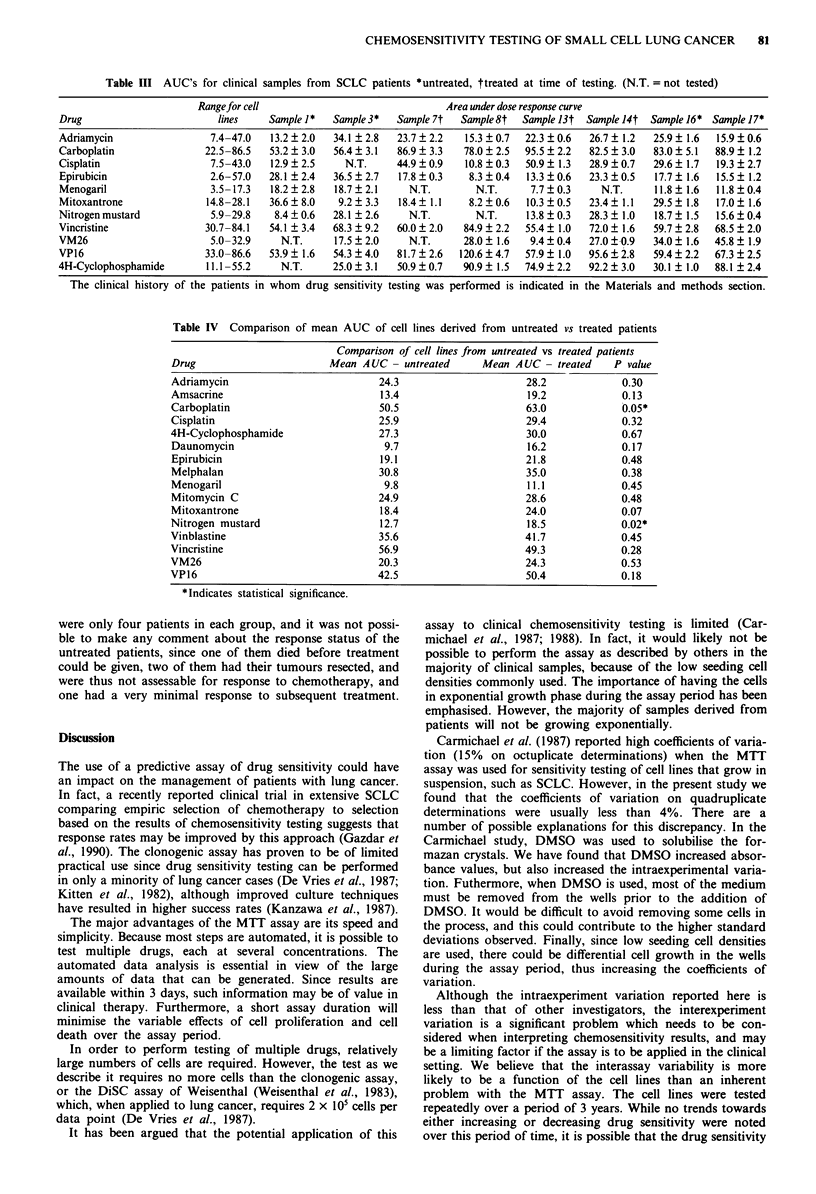

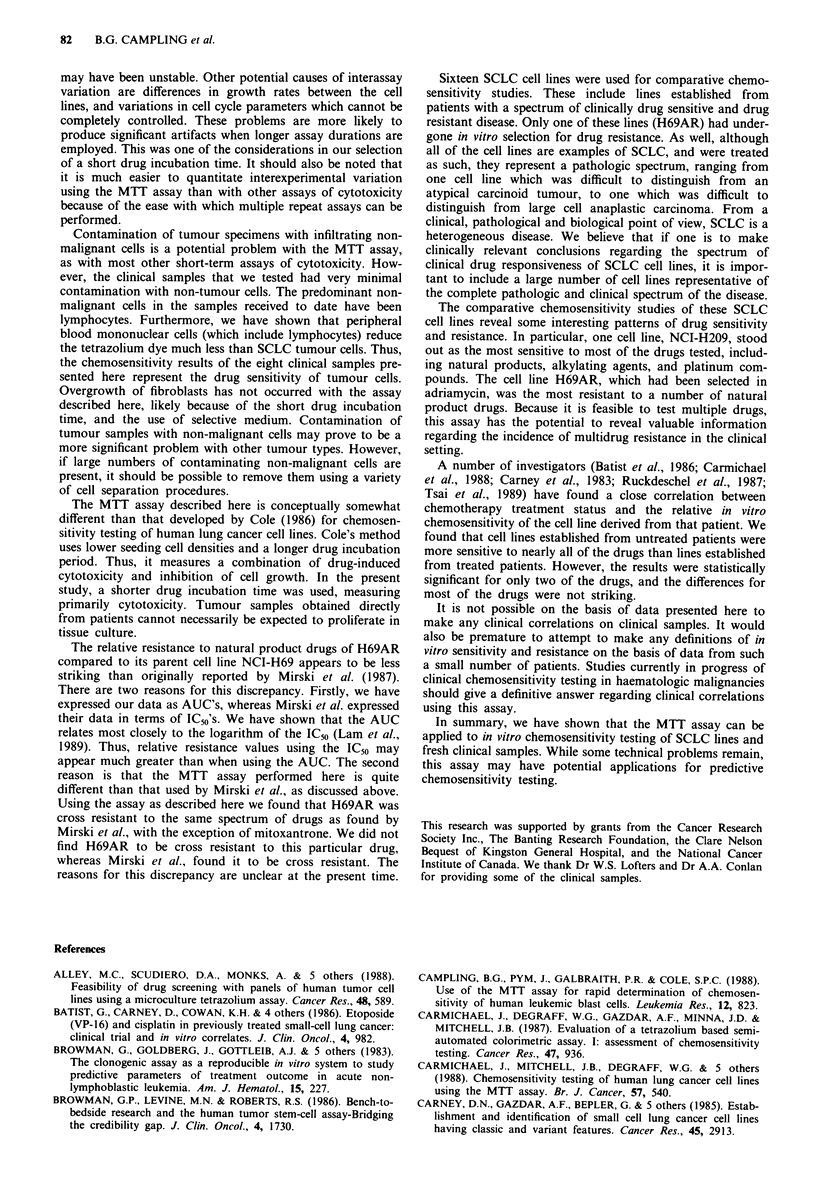

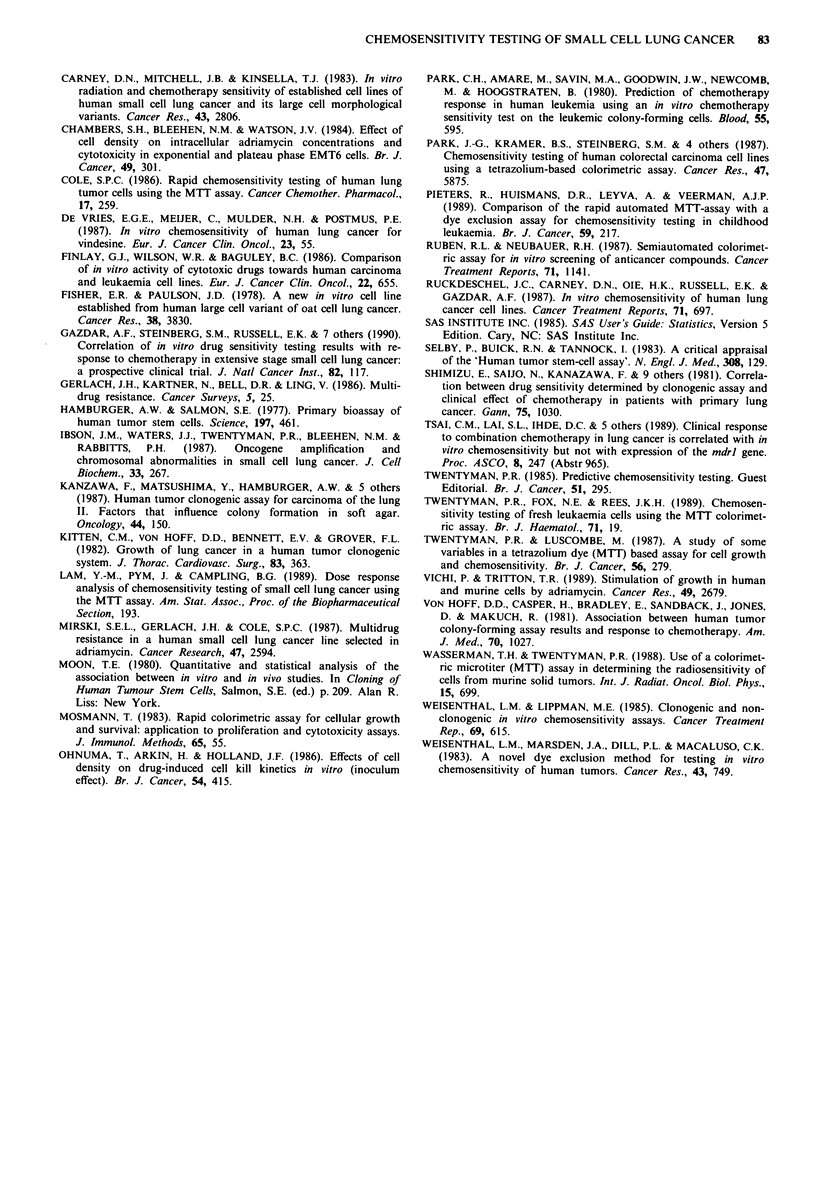


## References

[OCR_01477] Alley M. C., Scudiero D. A., Monks A., Hursey M. L., Czerwinski M. J., Fine D. L., Abbott B. J., Mayo J. G., Shoemaker R. H., Boyd M. R. (1988). Feasibility of drug screening with panels of human tumor cell lines using a microculture tetrazolium assay.. Cancer Res.

[OCR_01481] Batist G., Carney D. N., Cowan K. H., Veach S. R., Gilliom M., Bunn P. A., Ihde D. C. (1986). Etoposide (VP-16) and cisplatin in previously treated small-cell lung cancer: clinical trial and in vitro correlates.. J Clin Oncol.

[OCR_01492] Browman G. P., Levine M. N., Roberts R. S. (1986). Bench-to-bedside research and the human tumor stem-cell assay--bridging the credibility gap.. J Clin Oncol.

[OCR_01497] Campling B. G., Pym J., Galbraith P. R., Cole S. P. (1988). Use of the MTT assay for rapid determination of chemosensitivity of human leukemic blast cells.. Leuk Res.

[OCR_01501] Carmichael J., DeGraff W. G., Gazdar A. F., Minna J. D., Mitchell J. B. (1987). Evaluation of a tetrazolium-based semiautomated colorimetric assay: assessment of chemosensitivity testing.. Cancer Res.

[OCR_01507] Carmichael J., Mitchell J. B., DeGraff W. G., Gamson J., Gazdar A. F., Johnson B. E., Glatstein E., Minna J. D. (1988). Chemosensitivity testing of human lung cancer cell lines using the MTT assay.. Br J Cancer.

[OCR_01512] Carney D. N., Gazdar A. F., Bepler G., Guccion J. G., Marangos P. J., Moody T. W., Zweig M. H., Minna J. D. (1985). Establishment and identification of small cell lung cancer cell lines having classic and variant features.. Cancer Res.

[OCR_01519] Carney D. N., Mitchell J. B., Kinsella T. J. (1983). In vitro radiation and chemotherapy sensitivity of established cell lines of human small cell lung cancer and its large cell morphological variants.. Cancer Res.

[OCR_01525] Chambers S. H., Bleehen N. M., Watson J. V. (1984). Effect of cell density on intracellular adriamycin concentration and cytotoxicity in exponential and plateau phase EMT6 cells.. Br J Cancer.

[OCR_01531] Cole S. P. (1986). Rapid chemosensitivity testing of human lung tumor cells using the MTT assay.. Cancer Chemother Pharmacol.

[OCR_01536] De Vries E. G., Meijer C., Mulder N. H., Postmus P. E. (1987). In vitro chemosensitivity of human lung cancer for vindesine.. Eur J Cancer Clin Oncol.

[OCR_01541] Finlay G. J., Wilson W. R., Baguley B. C. (1986). Comparison of in vitro activity of cytotoxic drugs towards human carcinoma and leukaemia cell lines.. Eur J Cancer Clin Oncol.

[OCR_01546] Fisher E. R., Paulson J. D. (1978). A new in vitro cell line established from human large cell variant of oat cell lung cancer.. Cancer Res.

[OCR_01551] Gazdar A. F., Steinberg S. M., Russell E. K., Linnoila R. I., Oie H. K., Ghosh B. C., Cotelingam J. D., Johnson B. E., Minna J. D., Ihde D. C. (1990). Correlation of in vitro drug-sensitivity testing results with response to chemotherapy and survival in extensive-stage small cell lung cancer: a prospective clinical trial.. J Natl Cancer Inst.

[OCR_01557] Gerlach J. H., Kartner N., Bell D. R., Ling V. (1986). Multidrug resistance.. Cancer Surv.

[OCR_01561] Hamburger A. W., Salmon S. E. (1977). Primary bioassay of human tumor stem cells.. Science.

[OCR_01565] Ibson J. M., Waters J. J., Twentyman P. R., Bleehen N. M., Rabbitts P. H. (1987). Oncogene amplification and chromosomal abnormalities in small cell lung cancer.. J Cell Biochem.

[OCR_01571] Kanzawa F., Matsushima Y., Hamburger A. W., Ishihara J., Sasaki Y., Shimizu E., Eguchi K., Shinkai T., Saijo N., Miyazawa N. (1987). Human tumor clonogenic assay for carcinoma of the lung. II. Factors that influence colony formation in soft agar.. Oncology.

[OCR_01577] Kitten C. M., Von Hoff D. D., Bennett E. V., Grover F. L. (1982). Growth of lung cancer in a human tumor clonogenic system.. J Thorac Cardiovasc Surg.

[OCR_01588] Mirski S. E., Gerlach J. H., Cole S. P. (1987). Multidrug resistance in a human small cell lung cancer cell line selected in adriamycin.. Cancer Res.

[OCR_01593] Moon T. E. (1980). Quantitative and statistical analysis of the association between in vitro and in vivo studies.. Prog Clin Biol Res.

[OCR_01599] Mosmann T. (1983). Rapid colorimetric assay for cellular growth and survival: application to proliferation and cytotoxicity assays.. J Immunol Methods.

[OCR_01604] Ohnuma T., Arkin H., Holland J. F. (1986). Effects of cell density on drug-induced cell kill kinetics in vitro (inoculum effect).. Br J Cancer.

[OCR_01609] Park C. H., Amare M., Savin M. A., Goodwin J. W., Newcomb M. M., Hoogstraten B. (1980). Prediction of chemotherapy response in human leukemia using an in vitro chemotherapy sensitivity test on the leukemic colony-forming cells.. Blood.

[OCR_01616] Park J. G., Kramer B. S., Steinberg S. M., Carmichael J., Collins J. M., Minna J. D., Gazdar A. F. (1987). Chemosensitivity testing of human colorectal carcinoma cell lines using a tetrazolium-based colorimetric assay.. Cancer Res.

[OCR_01622] Pieters R., Huismans D. R., Leyva A., Veerman A. J. (1989). Comparison of the rapid automated MTT-assay with a dye exclusion assay for chemosensitivity testing in childhood leukaemia.. Br J Cancer.

[OCR_01628] Ruben R. L., Neubauer R. H. (1987). Semiautomated colorimetric assay for in vitro screening of anticancer compounds.. Cancer Treat Rep.

[OCR_01633] Ruckdeschel J. C., Carney D. N., Oie H. K., Russell E. K., Gazdar A. F. (1987). In vitro chemosensitivity of human lung cancer cell lines.. Cancer Treat Rep.

[OCR_01642] Selby P., Buick R. N., Tannock I. (1983). A critical appraisal of the "human tumor stem-cell assay".. N Engl J Med.

[OCR_01645] Shimizu E., Saijo N., Kanzawa F., Hoshi A., Eguchi K., Shinkai T., Tominaga K., Sasaki Y., Fujita J., Nomori H. (1984). Correlation between drug sensitivity determined by clonogenic cell assay and clinical effect of chemotherapy in patients with primary lung cancer.. Gan.

[OCR_01661] Twentyman P. R., Fox N. E., Rees J. K. (1989). Chemosensitivity testing of fresh leukaemia cells using the MTT colorimetric assay.. Br J Haematol.

[OCR_01666] Twentyman P. R., Luscombe M. (1987). A study of some variables in a tetrazolium dye (MTT) based assay for cell growth and chemosensitivity.. Br J Cancer.

[OCR_01657] Twentyman P. R. (1985). Predictive chemosensitivity testing.. Br J Cancer.

[OCR_01671] Vichi P., Tritton T. R. (1989). Stimulation of growth in human and murine cells by adriamycin.. Cancer Res.

[OCR_01675] Von Hoff D. D., Casper J., Bradley E., Sandbach J., Jones D., Makuch R. (1981). Association between human tumor colony-forming assay results and response of an individual patient's tumor to chemotherapy.. Am J Med.

[OCR_01681] Wasserman T. H., Twentyman P. (1988). Use of a colorimetric microtiter (MTT) assay in determining the radiosensitivity of cells from murine solid tumors.. Int J Radiat Oncol Biol Phys.

[OCR_01687] Weisenthal L. M., Lippman M. E. (1985). Clonogenic and nonclonogenic in vitro chemosensitivity assays.. Cancer Treat Rep.

[OCR_01692] Weisenthal L. M., Marsden J. A., Dill P. L., Macaluso C. K. (1983). A novel dye exclusion method for testing in vitro chemosensitivity of human tumors.. Cancer Res.

